# Pectoralis Major Myocutaneous Flap Reconstruction Following a Segmental Mandibulectomy for Advanced Oral Cavity Squamous Cell Carcinoma: A Case Report

**DOI:** 10.7759/cureus.108812

**Published:** 2026-05-13

**Authors:** Karim D García

**Affiliations:** 1 Surgery, Instituto Mexicano del Seguro Social, Puebla, MEX

**Keywords:** head and neck reconstruction, mandibular reconstruction, oral cavity cancer, pectoralis major myocutaneous flap, squamous cell carcinoma

## Abstract

Reconstruction of defects following ablative surgery for oral cavity cancer remains one of the most complex challenges in head and neck surgery. Although microvascular free flaps are currently considered the gold standard for mandibular reconstruction, regional pedicled flaps such as the pectoralis major myocutaneous (PMMC) flap continue to play an important role in selected patients.

We report the case of an 82-year-old female diagnosed with moderately differentiated squamous cell carcinoma of the inferior alveolar ridge (T4aN2bM0), who underwent segmental mandibulectomy with bilateral neck dissection, tracheostomy, and reconstruction using a left PMMC flap. The flap provided reliable soft tissue coverage and restoration of oral cavity continuity following tumor resection. This case highlights the continued relevance of the PMMC flap in modern head and neck reconstruction, particularly in elderly patients or those with comorbidities that limit the use of microvascular reconstruction.

## Introduction

Head and neck malignancies frequently require extensive surgical resections that produce complex composite defects involving bone, mucosa, skin, and soft tissues. Reconstruction following tumor ablation is critical to restore essential functions such as speech, swallowing, mastication, and airway protection, while also preserving facial aesthetics and enabling timely initiation of adjuvant oncologic therapy when required [1].

Mandibular defects represent a particular reconstructive challenge because the mandible plays a central role in maintaining occlusion, lower facial contour, and oral competence. Loss of mandibular continuity may result in dysphagia, impaired speech articulation, malocclusion, and severe cosmetic deformity [1].

Over the past decades, microvascular free tissue transfer has become the preferred reconstructive technique for mandibular defects. Free flaps such as the fibula osteocutaneous flap allow simultaneous reconstruction of bone and soft tissue, achieving excellent functional and aesthetic outcomes [2].

Nevertheless, microvascular reconstruction requires prolonged operative time, specialized surgical expertise, and favorable vascular conditions at both donor and recipient sites. Elderly patients or individuals with significant comorbidities may not be optimal candidates for such procedures.

The pectoralis major myocutaneous (PMMC) flap, first described by Ariyan in 1979, represented a milestone in head and neck reconstructive surgery and became the workhorse flap for reconstruction of oral cavity and pharyngeal defects prior to the widespread adoption of microvascular techniques [3].

The PMMC flap is based on the thoracoacromial artery and provides a large volume of well-vascularized muscle and skin that can be transferred reliably to the head and neck region without microvascular anastomosis [4]. Although its use has declined with the advancement of microsurgical reconstruction, the PMMC flap remains an important option in elderly patients, salvage procedures, and settings where microvascular reconstruction is not feasible [5].

This report describes the successful use of a PMMC flap for reconstruction following segmental mandibulectomy in an elderly patient with advanced oral cavity squamous cell carcinoma.

## Case presentation

An 82-year-old female presented to the head and neck surgery department with a two-month history of progressive enlargement of a lesion in the oral cavity. The patient denied any previous oncologic or surgical history. Her past medical history was notable for systemic arterial hypertension without pharmacological treatment.

**Figure 1:**
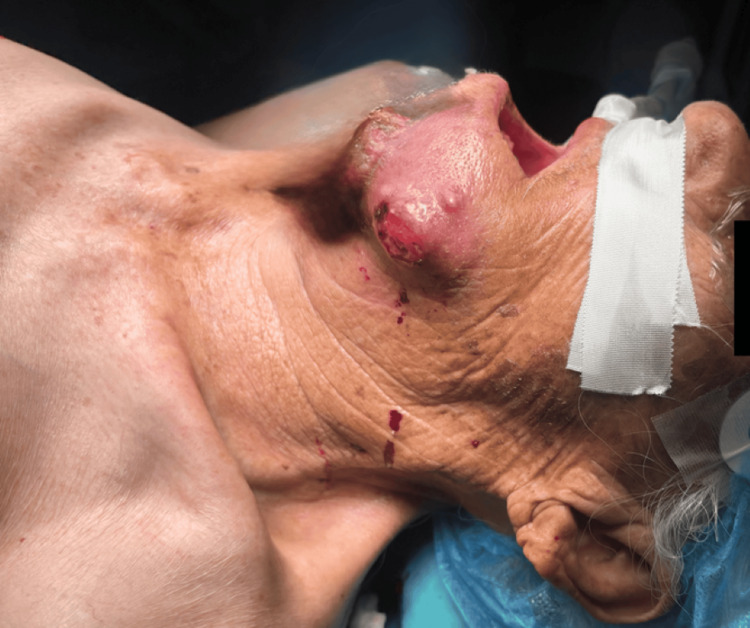
Preoperative clinical image of the oral cavity tumor Exophytic lesion involving the inferior alveolar ridge with extension to the mental region.

At the time of initial clinical evaluation, an exophytic lesion involving the inferior alveolar ridge with extension to the mental region was observed. The lesion showed progressive enlargement and ulceration of the overlying skin. A preoperative clinical photograph demonstrates the extent of the lesion involving the chin region (Figure 1).

An incisional biopsy performed by a referring physician revealed moderately differentiated squamous cell carcinoma.

Physical examination demonstrated an exophytic tumor measuring approximately 8 × 4 cm, located in the inferior alveolar ridge, with extension to the mental region and associated skin ulceration. Cervical examination revealed palpable lymphadenopathy in the submental and submandibular regions.

**Figure 2:**
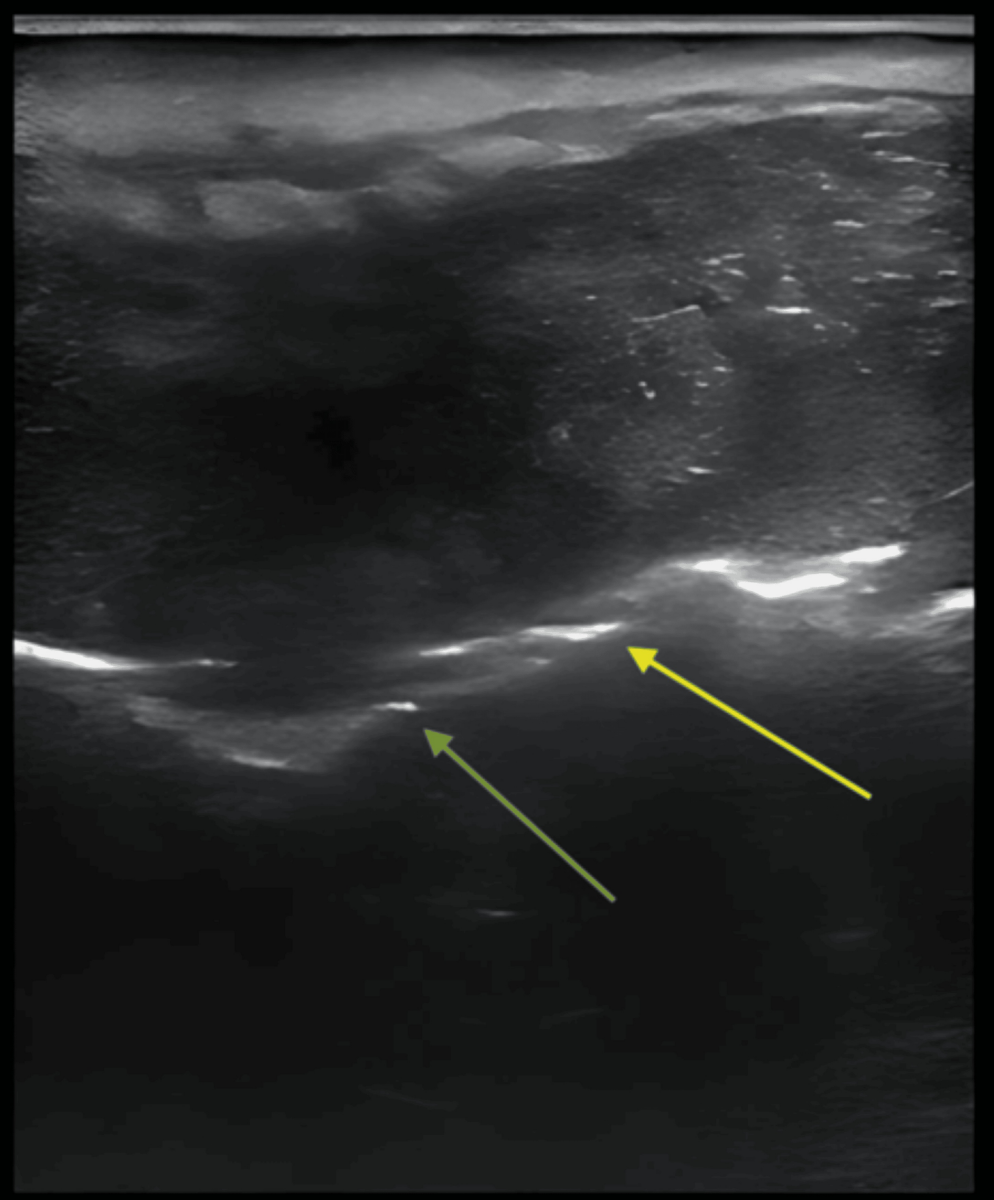
Ultrasound evaluation of oral cavity tumor with cortical bone involvement Ultrasound of the floor of the mouth demonstrates a hypoechoic infiltrative lesion involving the oral mucosa and adjacent soft tissues. The mandibular cortex is identified as a hyperechoic linear structure (yellow arrow), with focal irregularity suggestive of cortical bone invasion (green arrow).

Ultrasound performed on 02/25 demonstrated tumor involvement of the oral cavity and floor of the mouth, with loss of the interface between the mucosa of the lower lip and the underlying mandibular bone. Cervical lymph nodes with cortical thickening were identified at level IA (Figure 2). 

**Figure 3:**
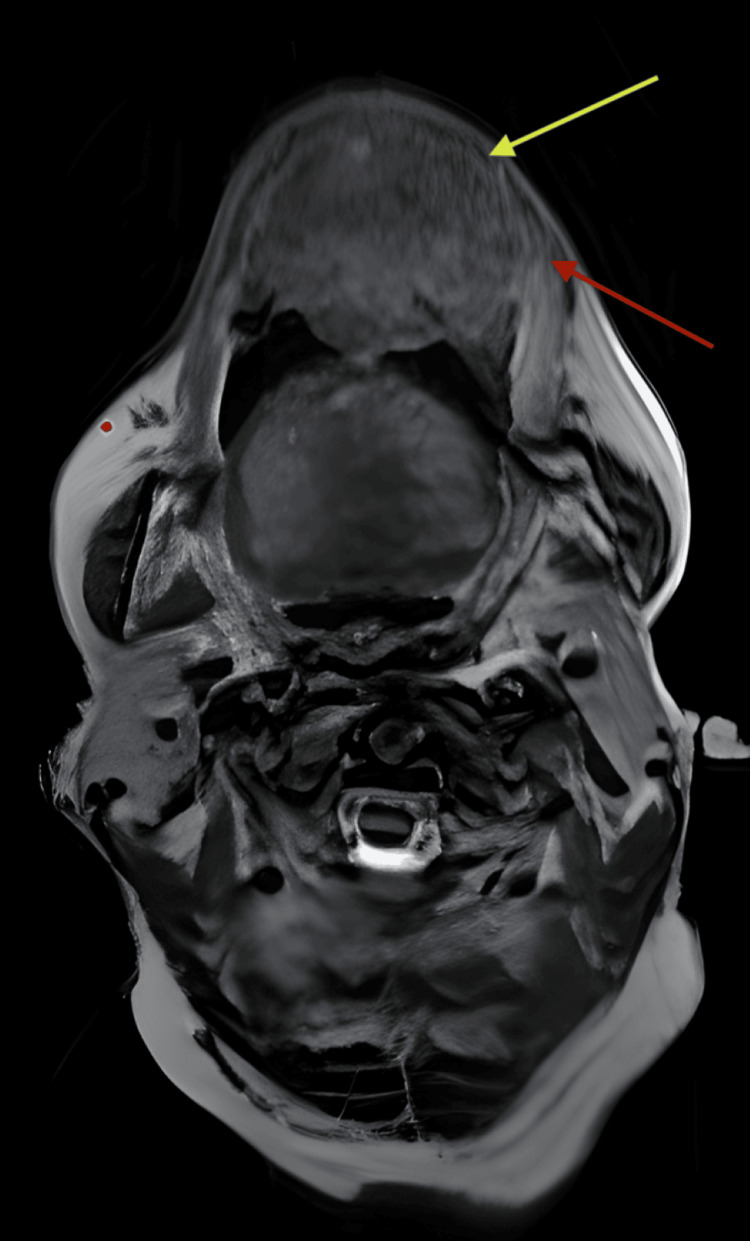
Contrast-enhanced MRI of an infiltrative mandibular alveolar ridge tumor Axial contrast-enhanced MRI shows a large, expansile lesion centered in the left mandibular alveolar ridge, extending across the midline into the right alveolar ridge. The mass demonstrates homogeneous enhancement, diffusion restriction on diffusion-weighted imaging (DWI) with corresponding low apparent diffusion coefficient (ADC) values, and measures approximately 62 × 44 mm (yellow arrow). There is associated cortical bone destruction with loss of mandibular continuity (red arrow). Adjacent soft tissue involvement is noted.

**Figure 4:**
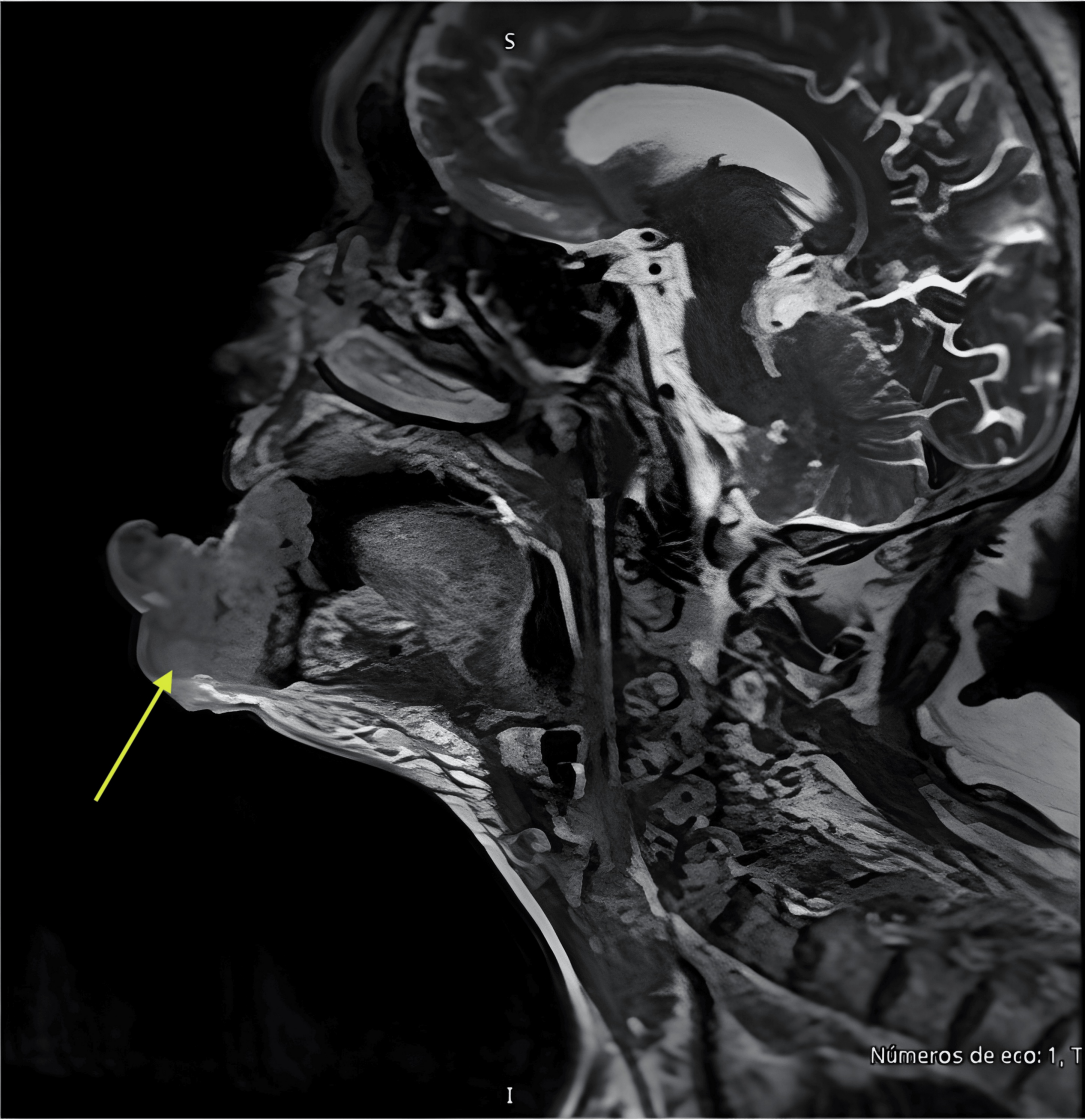
Sagittal MRI demonstrating locally advanced mandibular tumor with deep soft tissue extension Sagittal MRI demonstrates a large infiltrative lesion arising from the mandibular alveolar ridge, with extension into adjacent deep soft tissues of the floor of the mouth and submandibular region. The lesion exhibits heterogeneous signal intensity and mass effect, with distortion of normal anatomical planes (yellow arrow).

Magnetic resonance imaging revealed tumor extension into the anterior floor of the mouth, displacing the mobile tongue posteriorly without clear invasion of the tongue musculature (Figures 3-4).

Based on clinical and radiological findings, the tumor was staged as T4aN2bM0 oral cavity squamous cell carcinoma.

After multidisciplinary evaluation, surgical treatment was planned, consisting of segmental mandibulectomy, bilateral neck dissection (levels I-III), tracheostomy, and reconstruction with a left PMMC flap.

Following tumor resection, a PMMC flap was performed. A skin paddle was designed caudal and medial to the areola, incorporating the perforator from the third intercostal space of the internal mammary artery. The medialized design was selected to ensure a more reliable vascular supply, while the lateral margin was tailored according to the reconstructive requirements.

Flap elevation was subsequently carried out, preserving the superior third of the pectoralis major muscle to minimize donor-site deformity. Maximal skeletonization of the vascular pedicle - specifically the pectoral branch of the thoracoacromial vessels - was performed to increase pedicle length and enhance the arc of rotation. Efforts were made to preserve the lateral thoracic artery by dividing the pectoralis minor muscle, thereby improving distal flap perfusion.

Finally, the flap was transferred through a submuscular tunnel over the clavicle and inset in the supraclavicular region to provide coverage of the intraoral defects.

**Figure 5:**
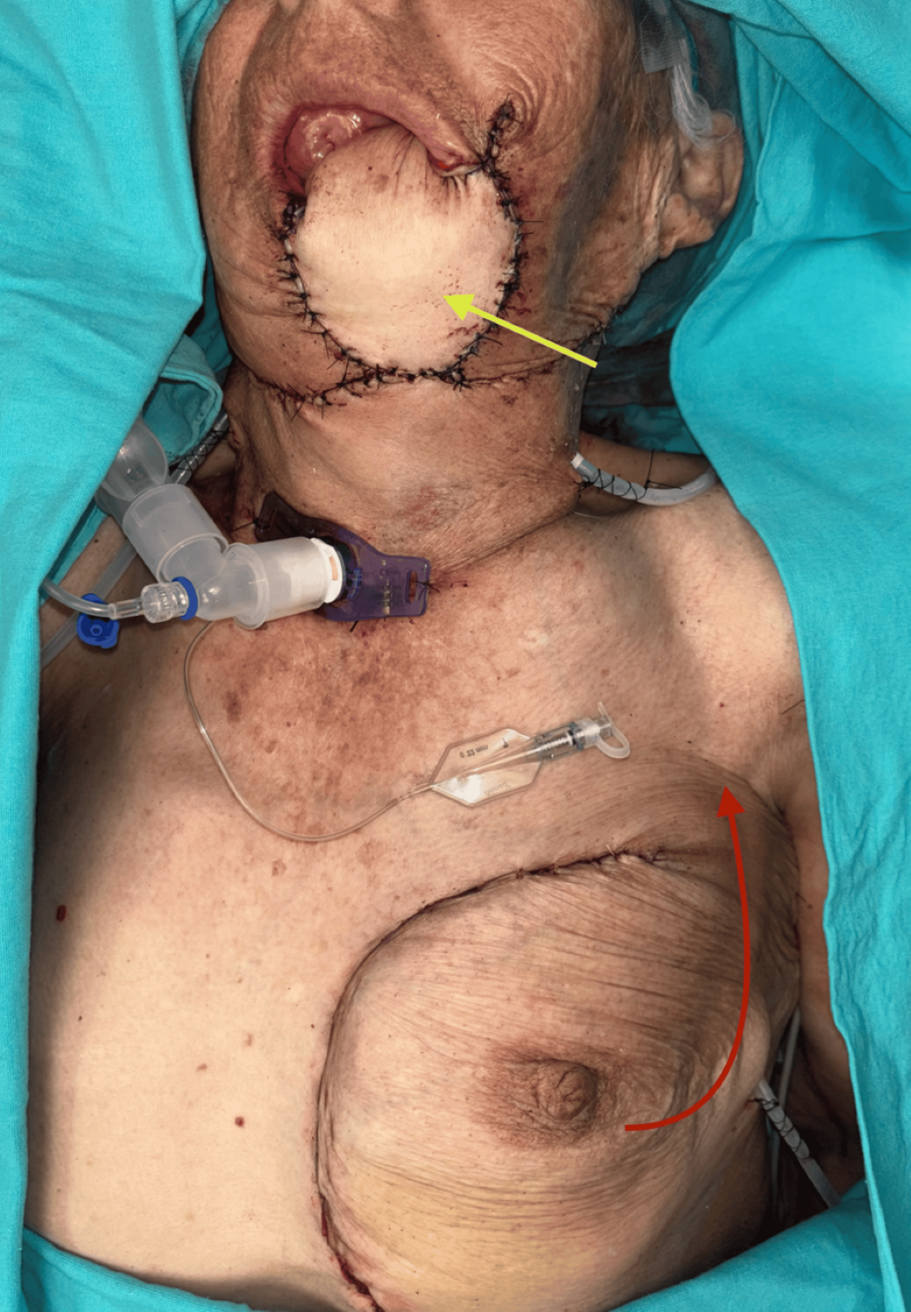
Pectoralis major myocutaneous flap Intraoperative image showing reconstruction of the oral cavity defect using a pectoralis major myocutaneous flap, with the donor site located on the anterior chest wall. The skin paddle is oriented to line the intraoral defect (yellow arrow), while the vascular pedicle (red arrow), based on the thoracoacromial vessels, is preserved and tunneled into the neck to ensure adequate perfusion.

An intraoperative image demonstrates the reconstructed defect using the PMMC flap, as well as the donor-site incision on the anterior chest wall (Figure 5).

The decision not to perform bony reconstruction was based on an individualized risk-benefit assessment. The patient was of advanced age and presented with significant frailty, which increased the risk of prolonged anesthesia, perioperative complications, and postoperative morbidity associated with more complex reconstructive procedures such as osseous free flaps.

The patient demonstrated a favorable postoperative course, with no clinical evidence of flap congestion, venous compromise, or ischemia during the immediate postoperative period. Wound healing progressed appropriately, without signs of infection, dehiscence, or partial flap loss. Oral intake was gradually reintroduced and well-tolerated, with acceptable functional adaptation given the extent of resection.

The patient has remained under close outpatient follow-up, with regular clinical evaluations focused on flap viability, wound integrity, and functional status, including speech and swallowing. Surveillance imaging has been performed at scheduled intervals, with no radiologic evidence of local recurrence or distant metastasis to date. Overall, the patient maintains a stable clinical condition, with no complications reported during follow-up.

## Discussion

Reconstruction of head and neck defects following oncologic resection is essential to restore both functional capacity and quality of life. Mandibular reconstruction plays a critical role in preserving mastication, swallowing, speech articulation, and facial symmetry [1].

Microvascular free flaps are currently considered the gold standard for mandibular reconstruction because they allow restoration of bone continuity and provide excellent functional outcomes [2]. However, these procedures require prolonged operative times and specialized microsurgical expertise.

The PMMC flap remains one of the most reliable regional flaps in head and neck reconstruction. Its predictable vascular supply from the thoracoacromial artery and technical simplicity allow rapid harvest and transfer without the need for microvascular anastomosis [4].

Liu et al. reported a clinical series of 118 PMMC flaps, demonstrating low complication rates and high flap survival, particularly in elderly patients or those with compromised vascular status [5].

Onoda et al. described an extended PMMC flap incorporating additional vascular supply from the lateral thoracic vessels, improving perfusion and expanding reconstructive versatility [6].

Furthermore, modifications such as the midline sternal skin paddle design described by Oh et al. have been proposed to reduce donor-site deformity and improve aesthetic outcomes, particularly in female patients [7].

Although PMMC flaps may produce more bulk compared with free flaps and may be associated with donor-site morbidity, their reliability and shorter operative time make them valuable reconstructive options in selected patients.

In the present case, the patient’s advanced age and clinical status favored the use of a regional pedicled flap. The PMMC flap provided adequate soft tissue coverage and allowed satisfactory postoperative recovery.

## Conclusions

The PMMC flap remains a reliable and versatile reconstructive option for head and neck defects following oncologic resection. Although microvascular free flaps represent the gold standard for mandibular reconstruction, the PMMC flap continues to play an important role in selected patients. This case demonstrates the successful use of the PMMC flap for reconstruction following segmental mandibulectomy in an elderly patient with advanced oral cavity cancer.
